# Field study on compaction effect and energy evolution characteristics of highway subgrade under low-amplitude vibration

**DOI:** 10.1371/journal.pone.0361901

**Published:** 2026-10-29

**Authors:** Xiaobo Liu, Ping Xie, Wubin Wang

**Affiliations:** 1 Sichuan Road & Bridge (Group) Co. Ltd., Chengdu, China; 2 School of Civil Engineering, Southwest Jiaotong University, Chengdu, China; University of Sharjah, UNITED ARAB EMIRATES

## Abstract

Subgrade compaction is a critical procedure in highway construction. To elucidate the dynamic characteristics and energy transfer mechanisms throughout the entire compaction process, this study utilized four test lanes to conduct field experiments on highway subgrade under varying numbers of rolling passes. Comprehensive measurements and analyses were performed regarding the dynamic characteristics of vibration waves, energy distribution, and the evolution of soil compactness during operation. Furthermore, the compaction efficiency and energy transfer within the subgrade were quantitatively evaluated. The results indicate that the dynamic behavior of subgrade materials varies significantly across different compaction stages, which are consequently categorized into three distinct phases: loose, optimal compaction, and over-compaction. In this experiment, the third rolling pass represented the optimal compaction state, characterized by distinct resonance peaks, a regular frequency spectrum, and highly efficient energy transfer. As compactness increases, the horizontal propagation of vibration waves strengthens, whereas vertical attenuation accelerates, causing energy to concentrate beneath the vibration source. Additionally, the proposed method for evaluating compaction efficiency based on the energy transfer coefficient corroborates the three-stage evolution of the process, offering a direct, quantitative indicator for real-time quality assessment and endpoint determination. These findings provide new theoretical insights and empirical data to support intelligent and refined process control in subgrade compaction.

## 1 Introduction

By the end of 2024, the total length of China’s expressway network had reached 190,700 kilometers. The surge in travel demand and population mobility has introduced new challenges to the national road transport system, necessitating higher standards for highway construction quality [1–3]. As a foundational component of this system, the subgrade bears its self-weight, the pavement structure, and dynamic traffic loads [4,5]. Consequently, subgrade compaction is a critical phase in road engineering, as it dictates the infrastructure’s long-term performance and service life [6,7]. Traditional quality control, however, relies heavily on empirical judgment and post-process spot checks, such as the sand cone method [8,9]. This approach is limited by time lags and the discrete nature of sampling, which precludes real-time optimization or closed-loop control. These limitations increase the risk of under- or over-compaction, potentially resulting in structural defects or resource wastage [10–12]. Therefore, the implementation of intelligent and refined process control in highway subgrade compaction is essential [13,14].

Current research on highway subgrade compaction primarily focuses on compaction mechanisms [15], quality standards [16], process optimization, numerical simulations [17], and prediction models [18,19]. Specifically, investigations into compaction mechanisms aim to elucidate the densification behavior of subgrade materials under external loads, providing a theoretical basis for optimizing compaction technologies. Regarding quality control, conventional assessment methods such as the sand cone test [18], dynamic cone penetrometer [20,21], water replacement [22,23], and California Bearing Ratio (CBR) [24] tests are labor-intensive and limited by discrete, sparse sampling. In contrast, modern techniques like the Portable Falling Weight Deflectometer (PFWD) and GeoGauge facilitate rapid, non-destructive evaluation [25, 26]. Furthermore, Intelligent Compaction (IC) has emerged as a leading trend in process development. By utilizing accelerometers and GPS sensors installed on the roller’s steel drum, it acquires real-time information such as the vibratory drum response, number of rolling passes, and position, thereby realizing the integration of “compaction-detection-feedback” [27–31]. In the realm of simulation, the Discrete Element Method (DEM) is extensively utilized to model the particle-scale movement and force chain evolution of granular materials [32,33]. Finally, artificial intelligence algorithms are increasingly integrated into prediction models to forecast construction parameters and quality, thereby optimizing construction strategies [34–36].

Due to the inherent complexity and time constraints associated with field testing, the majority of research relies on numerical simulation methods, resulting in a scarcity of empirical in-situ data [4]. However, compaction quality is fundamentally governed by the properties of the subgrade fill material and the roller control parameters [37,38]. Unlike railway construction, which utilizes standardized Group A and B fillers, highway subgrades typically employ locally sourced materials that exhibit significant anisotropy and heterogeneity [39]. Consequently, numerical simulations and theoretical models often fail to accurately characterize the dynamic evolution of material parameters under actual engineering conditions.

To this end, this study conducted a field experimental study on subgrade compaction performance, based on a highway construction site currently underway. By subjecting four planned lanes to varying numbers of rolling passes, the full-process acceleration response characteristics of both the subgrade interior and the vibratory roller drum were acquired. The study aims to reveal the dynamic response characteristics of vibration waves and the energy transfer mechanisms during the highway subgrade compaction process, thereby providing insights for the design of intelligent and refined process control for subgrade compaction.

## 2 Field compaction tests for subgrade

### 2.1 Project overview

The field tests were conducted on a subgrade section of the Tongzi Expressway, currently under construction in Sichuan Province. As illustrated in Fig 1, a test area measuring 40 m × 20 m was established. The subgrade comprises a soil-rock mixture, utilizing fill material composed of locally sourced weathered sandstone and soil particles. This filler is characterized by a predominance of medium-to-coarse grains, exhibiting angular to sub-angular shapes and moderate gradation. The material possesses a natural density of approximately 2.5–2.7 g/cm^3^ and a porosity of 10%–20%; furthermore, its moderate-to-high permeability facilitates efficient water drainage. The particle size of the fill material ranges from 0 to 60 mm, and the field-measured moisture content is approximately 7% to 11%. According to the compaction test results, the optimum moisture content of the fill is 8.8%, the maximum dry density is 2 g/cm^3^, the uniformity coefficient Cu = 29.3, and the coefficient of curvature Cc = 1.0.

**Fig 1:**
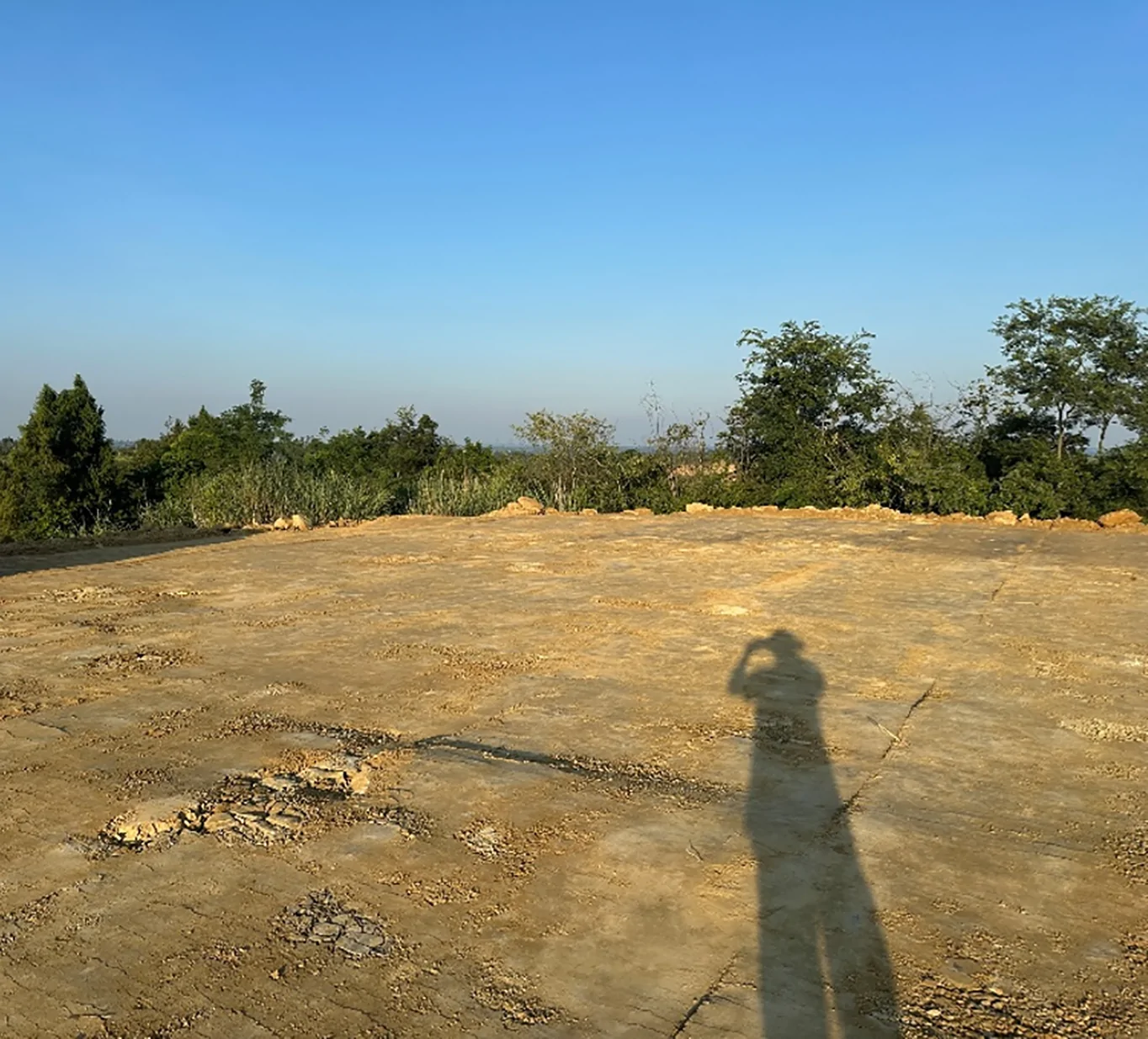
Field test site.

### 2.2 Test equipment

The construction process for highway subgrade filling encompasses the transportation and spreading of fill material, followed by surface leveling and compaction. Among these stages, subgrade compaction is critical to the integrity of the construction. For this study, the XCMG XS263J vibratory roller was selected as the primary compaction equipment; its key technical specifications are presented in Table 1.

**Table 1:** Technological parameters of rollers.

Technical parameters	Values
Work mass of vibratory roller	26000 kg
Vibratory wheel width	2170 mm
Vibratory wheel diameter	1600 mm
High-amplitude vibration frequency	27 Hz
Low-amplitude vibration frequency	32 Hz
High excitation force	405 kN
Low excitation force	290 kN
High-amplitude vibration value	1.9 mm
Low-amplitude vibration value	0.95 mm

Data acquisition was performed using a Donghua Testing DH5922D 32-channel dynamic system. Two models of acceleration sensors were employed: the Donghua Testing 1C301 (±16 g range), which was mounted on the vibrating drum, and the 1C302 (±5 g range), which was embedded within the subgrade fill. It should be noted that if subjected to accelerations beyond their specified ranges, the sensors continue to provide signal output, albeit with a potential slight degradation in linearity. For the experimental analysis, a coordinate system was defined wherein the x-axis corresponds to the roller’s forward travel, the y-axis represents the transverse direction perpendicular to movement, and the z-axis denotes the vertical direction relative to the subgrade surface. To maintain sensor orientation and prevent rotation during operation, a lightweight, high-stiffness square plate was attached to the base of each sensor.

### 2.3 Monitoring point layout

To evaluate the dynamic response of the mixed fill material, four identical test lanes were constructed at the site, as illustrated in Fig 2. Each lane measured 30 m in length and 2.2 m in width, with a loose lift thickness of 0.50 m. Five accelerometers were embedded along each lane at 2 m intervals and a depth of 0.10 m. This configuration enabled the acquisition of surface vibration signals, thereby facilitating an analysis of the horizontal propagation characteristics of the vibration waves. Additionally, an accelerometer was mounted on the roller drum to characterize the energy coupling interaction between the drum and the subgrade.

**Fig 2:**
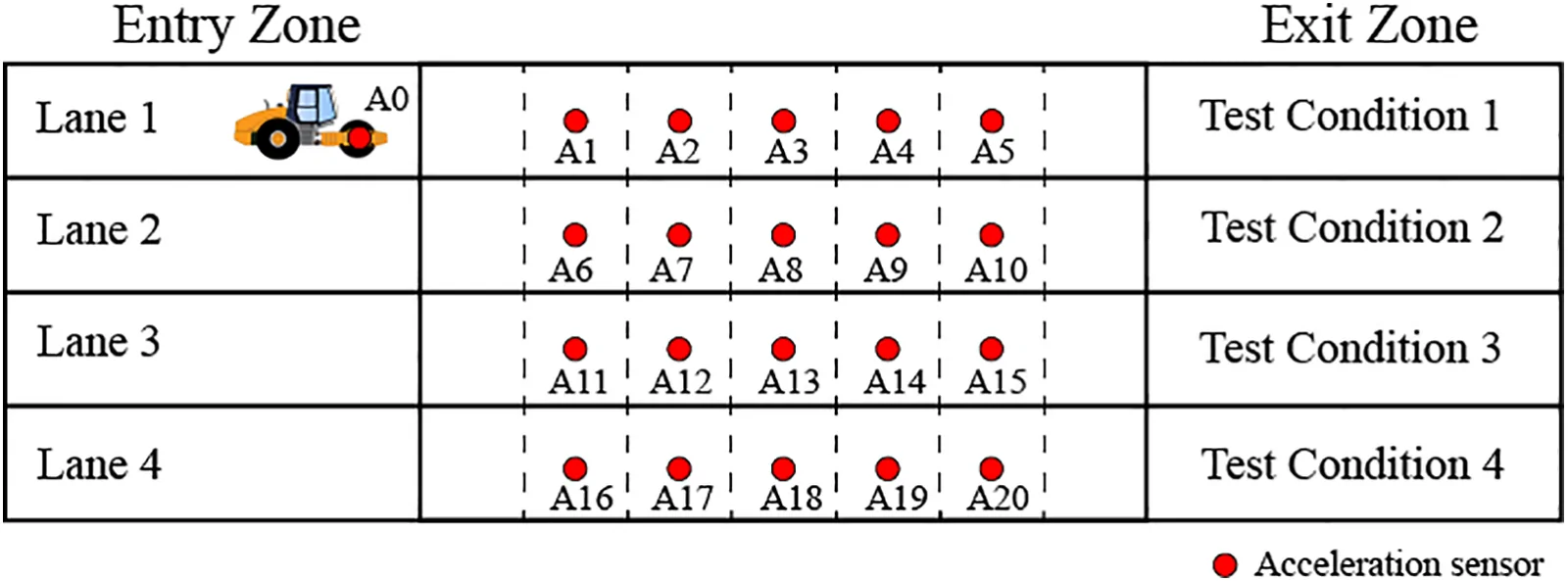
Lane demarcation and measurement points.

### 2.4 Test plan

Before testing, the fill material was placed and subjected to a single static rolling pass. To investigate the dynamic characteristics and the influence of pass count on subgrade compaction, four operating conditions were established, as detailed in Table 2. A single rolling pass was defined as one forward and reverse traversal of the lane at a speed of 3 km/h using low-amplitude vibration. Data acquisition was initiated at a sampling frequency of 1000 Hz before roller movement and continued uninterrupted until the experiment concluded. Following the tests, the sand cone method was employed to measure the compactness adjacent to the accelerometer locations.

**Table 2:** Test conditions.

No.	Vehicle speed	Vibration type	Roller pass count
1	3 km/h	Low-amplitude	1
2	3 km/h	Low-amplitude	2
3	3 km/h	Low-amplitude	3
4	3 km/h	Low-amplitude	4

## 3 Experimental results

### 3.1 Dynamic characteristics of vibration waves

#### 3.1.1 Time-domain characteristics.

To investigate the acceleration response of the subgrade surface during compaction, acceleration time-history curves were plotted for the two primary axes (X-direction and Z-direction), as presented in Figs 3 and 4. Note that certain data points are absent due to sensor malfunction. These time-histories clearly delineate the roller’s travel path. The initial sensor on the lane registers the acceleration amplitude first, followed sequentially by the subsequent sensors. Upon completion of the forward pass, the roller reverses direction, causing the final sensor to register the initial amplitude response for the return pass.

**Fig 3:**
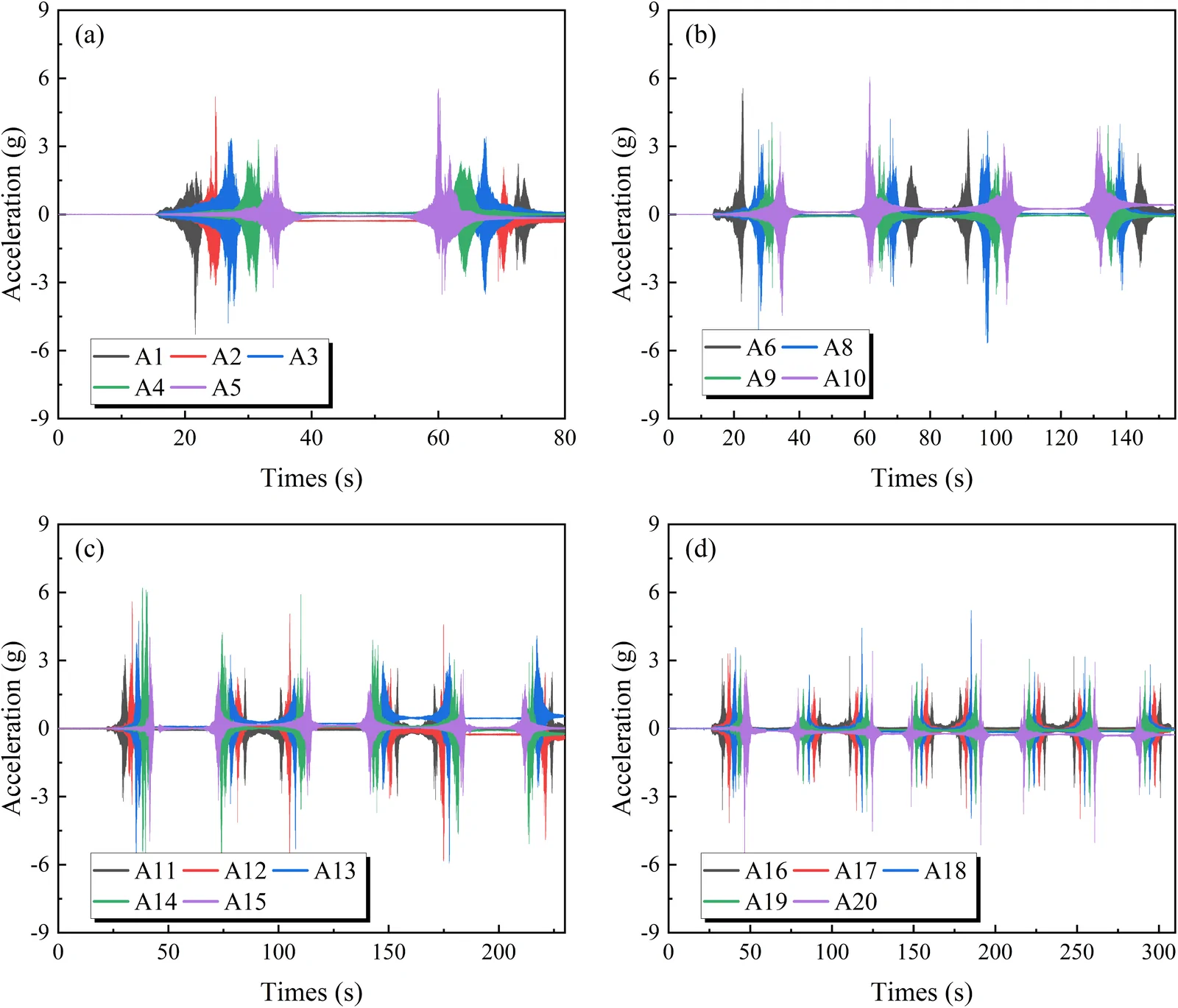
Time-history curves of acceleration in the X-direction for the subgrade under (a) 1 rolling pass, (b) 2 rolling passes, (c) 3 rolling passes, and (d) 4 rolling passes.

**Fig 4:**
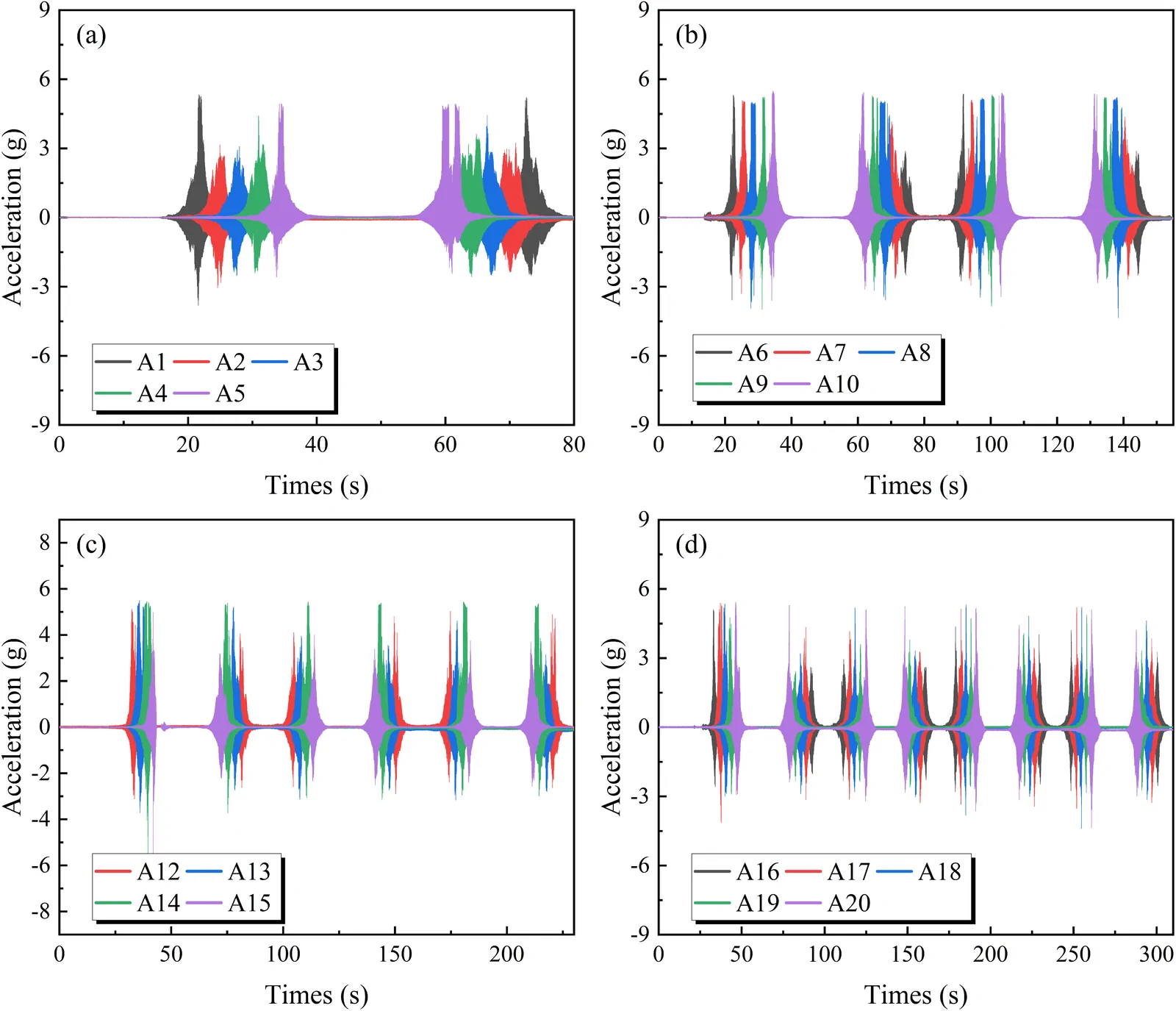
Time-history curves of acceleration in the Z-direction for the subgrade under (a) 1 rolling pass, (b) 2 rolling passes, (c) 3 rolling passes, and (d) 4 rolling passes.

Compared to the X-direction, the subgrade acceleration response in the Z-direction exhibits a more stable amplitude, typically fluctuating around 5g. Conversely, while peak amplitudes in the X-direction are generally lower in magnitude, the behavior is more complex and prone to sporadic extreme values. This disparity is likely attributed to the vertical application of the excitation force, which induces particle rearrangement, densification, and air expulsion. This relatively uniform process facilitates consistent energy transmission and a stable acceleration response. In contrast, X-direction acceleration arises from the coupling of multiple factors rather than a direct excitation source. Specifically, the roller’s advancement generates horizontal forces at the contact interface due to friction. Furthermore, traversing surface irregularities induces transient, high-frequency horizontal impacts, resulting in instantaneous acceleration peaks that significantly exceed the mean level. Consequently, the X-direction signal is characterized by frequent spikes and a stochastic peak distribution. Thus, the Z-direction response reflects the subgrade’s overall compaction stiffness and energy input, whereas the X-direction response indicates surface roughness, local material inhomogeneity, and complex dynamic coupling effects.

The peak amplitude of the acceleration signal reflects the magnitude of the subgrade’s dynamic response to the roller. To investigate the vibration response characteristics of the subgrade filler under varying numbers of rolling passes, the evolution of peak acceleration was analyzed, as illustrated in Fig 5. As the number of passes increases, the peak acceleration in both the X and Z directions exhibits a trend of initial decrease followed by an increase; this pattern is notably more pronounced in the Z-direction. This behavior suggests that during the initial compaction stage, the loose filler contains significant void space. Consequently, the energy supplied by the vibratory roller is primarily consumed in overcoming inter-particle friction and interlocking forces, facilitating particle rearrangement and void filling. This process results in substantial deformation and energy absorption, yielding a high response amplitude but low compaction efficiency. Conversely, in the later stages, once the material achieves a dense state with minimal potential for further particle movement or compression, energy is no longer effectively utilized for densification. Instead, the formation of a stable skeletal structure amplifies the acceleration response.

**Fig 5:**
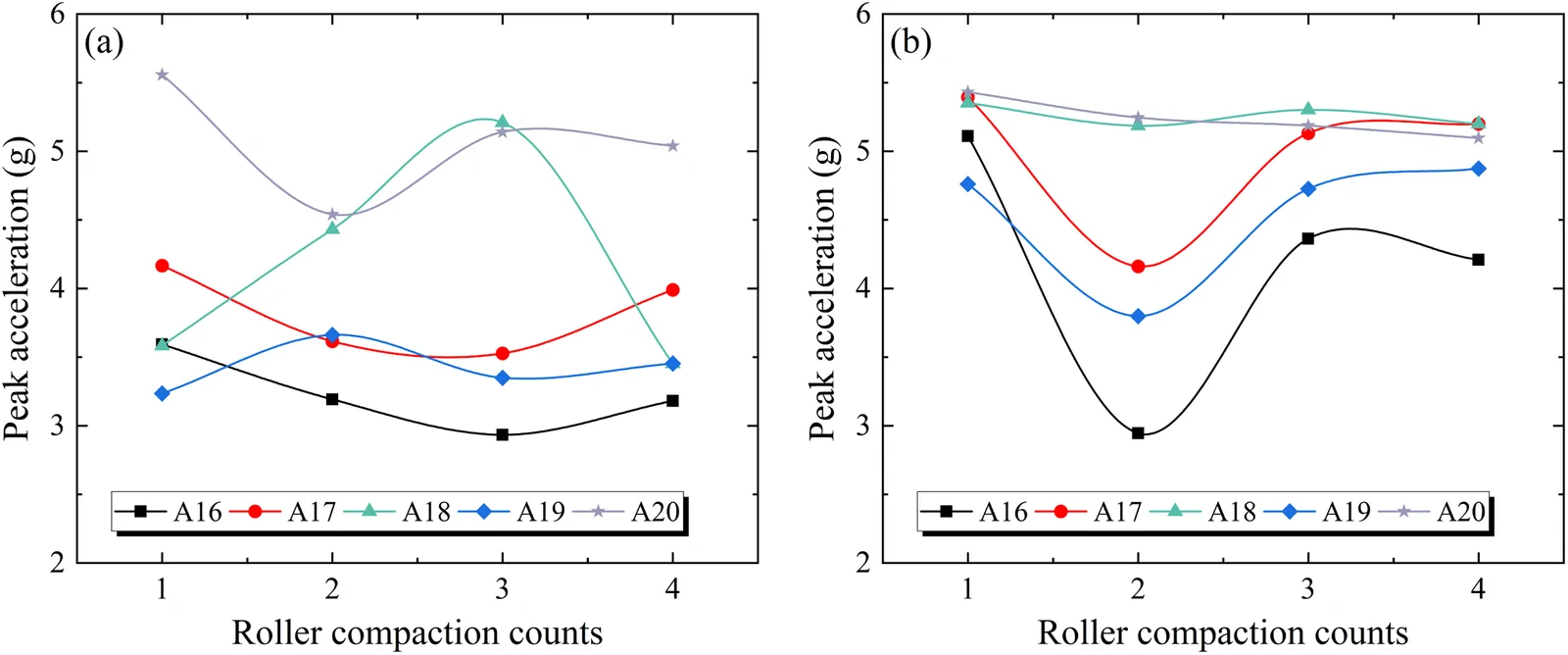
Peak accelerations in the (a) X-direction and (b) Z-direction of the subgrade under different numbers of passes in Condition 4.

#### 3.1.2 Frequency-domain characteristics.

The acceleration time-history data from the interior of the subgrade material at the middle position under Condition 4 across different passes were selected and subjected to a Fourier transform to obtain their frequency-domain response characteristics, as illustrated in Fig 6. Meanwhile, the corresponding Fourier spectral characteristics of the roller’s vibrating drum are plotted in Fig 7. Within the subgrade, the spectral curve morphologies in the X and Z directions are fundamentally consistent, differing primarily in amplitude. At the dominant frequency, the Z-direction amplitude generally exceeds that of the X-direction, a disparity that becomes more pronounced during the 3rd and 4th passes. As the number of passes increases, the peak amplitude in the Z-direction initially rises before decreasing at the 4th pass; conversely, the X-direction peak increases initially but begins to decline by the 3rd pass. Furthermore, while the spectral curves for the 1st and 2nd passes exhibit significant interference waves and harmonics, those for the 3rd and 4th passes appear more regular and stable. During the 3rd and 4th passes, the fundamental wave is located near 29 Hz, which is close to the frequency of the roller’s weak vibration mode. The second harmonic is located near 57 Hz, and the third harmonic near 85 Hz; the frequencies of the respective harmonics are essentially multiples of the fundamental frequency. Regarding the roller’s vibrating drum, the morphologies of the spectral curves in the X and Z directions during the same pass are also fundamentally consistent, with the Z-direction amplitude being slightly higher than that of the X-direction. As the number of rolling passes increases, both the dominant frequency and the amplitude of the vibrating drum continuously rise; however, the rate of this increase gradually diminishes, with the values at the 4th pass remaining nearly identical to those at the 3rd pass.

**Fig 6:**
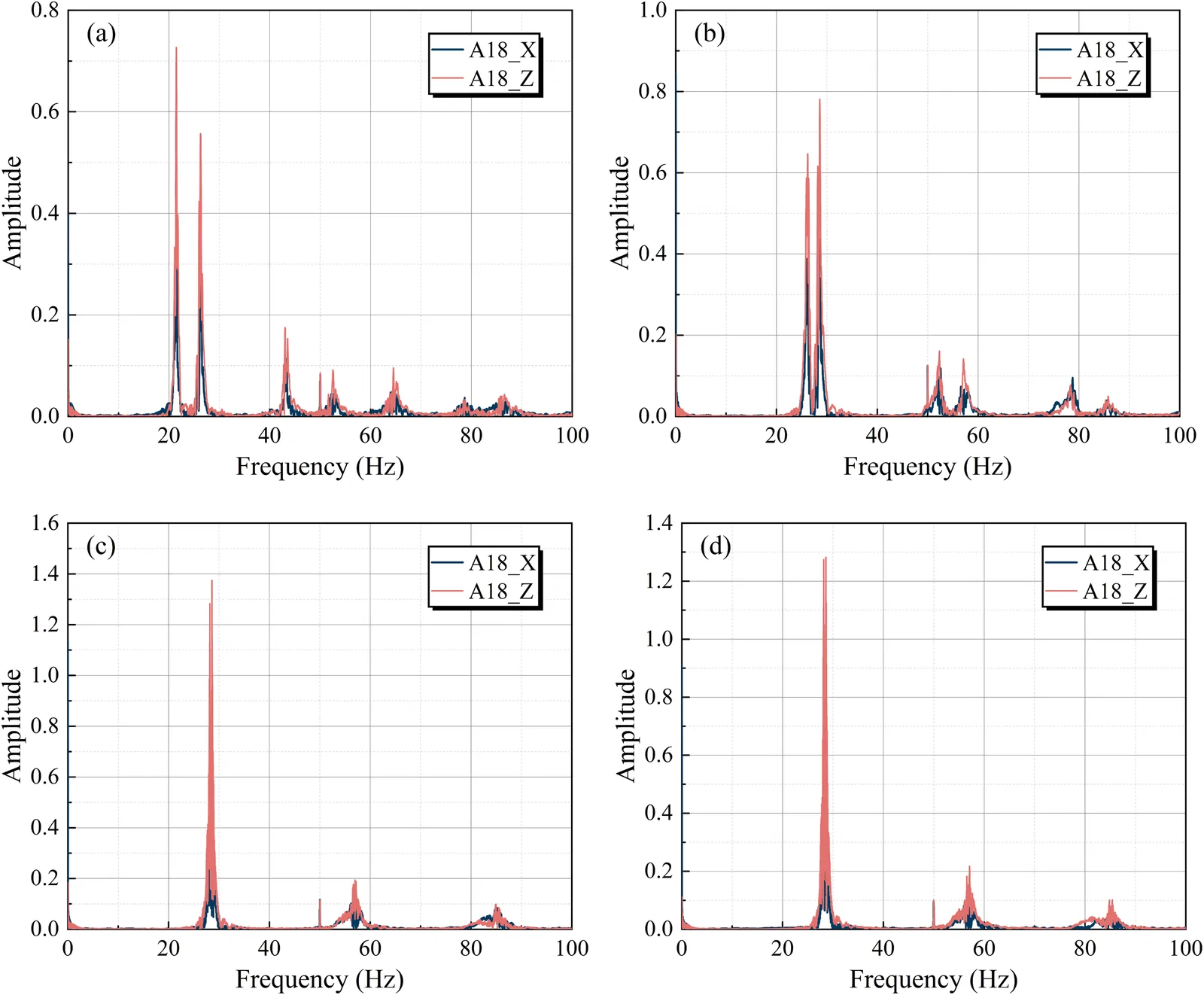
Fourier spectrum curves of the subgrade under (a) 1 pass, (b) 2 passes, (c) 3 passes, and (d) 4 passes of roller compaction.

**Fig 7:**
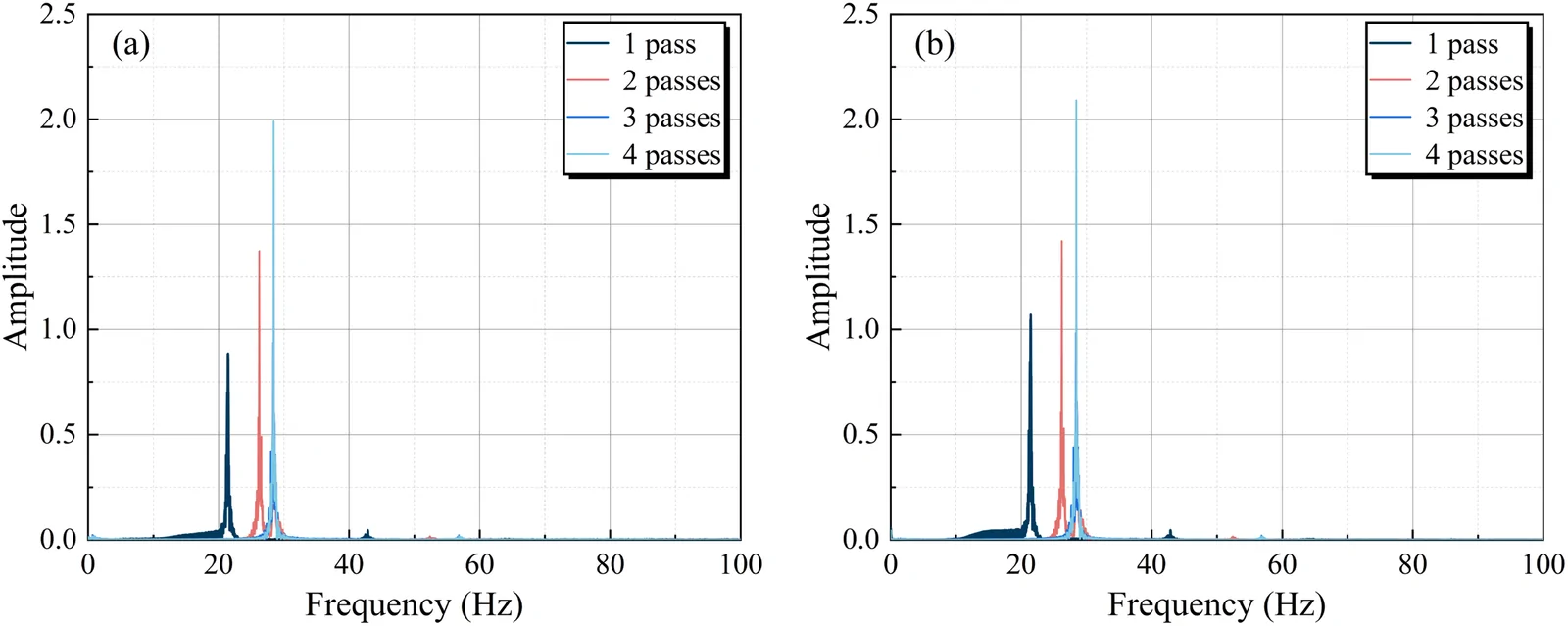
Fourier spectrum curves of the vibrating drum of road rollers in (a) the X direction and (b) the Z direction.

These observations indicate that the vibration responses in the X and Z directions share a common origin, primarily stemming from the forced vibration of the roller’s drum. As rolling progresses, the subgrade material transitions significantly from a loose granular mass to a quasi-elastic foundation. Consequently, the initially chaotic spectrum stabilizes as the material achieves greater uniformity and density, evidenced by the emergence of distinct harmonics based on the roller’s weak vibration frequency (29 Hz). This suggests the subgrade has evolved into a vibration system with a strong linear response. Notably, during the 3rd pass, the system stiffness appears to reach an optimum level where the natural frequency approaches the excitation frequency, triggering resonance. This phenomenon causes the Z-direction response amplitude to peak, signifying the optimal compaction effect. With continued compaction, the dominant frequency and amplitude of the roller’s drum remain virtually unchanged. The subsequent decrease in the Z-direction response, following the earlier decline in the X-direction, serves as a joint indicator that over-compaction may have induced microscopic degradation of internal stiffness or altered shear characteristics. This dynamic evolution demonstrates that monitoring the regularization of the vibration response spectrum and the inflection points of peak amplitudes allows for the precise determination of the optimal compaction state and the appropriate endpoint for construction.

By synthesizing time-domain and frequency-domain characteristics, the dynamic evolution of subgrade compaction can be summarized as follows: (1) Passes 1 and 2: The material is loose and heterogeneous, resulting in significant energy dissipation. Consequently, the spectrum appears chaotic, while the response amplitude undergoes a phase of accumulation. The primary objective during this stage is void reduction and initial densification. (2) Pass 3: The material achieves a high degree of homogeneity and optimal density, corresponding to maximum stiffness. As the system’s natural frequency approaches the excitation frequency, resonance occurs. This leads to a peak in the Z-direction response amplitude and the emergence of a clean, regular spectrum with distinct harmonics. At this juncture, the subgrade functions as a stable elastic foundation, signifying maximum compaction efficiency and optimal quality. (3) Pass 4: Continued rolling beyond this point means energy input is no longer effectively utilized for densification. The Z-direction response decreases, following an earlier decline in the X-direction response. Although the spectrum remains regular, the amplitude reduction suggests the onset of microscopic internal degradation. Further rolling results in energy waste and potential detriment to long-term performance.

### 3.2. Energy characteristics of vibration waves

#### 3.2.1 Time-frequency domain characteristics of energy.

To investigate the energy response characteristics during the roller compaction process, the Hilbert spectrum characteristics in two directions were plotted using the Hilbert-Huang Transform (HHT), a three-dimensional time-frequency-amplitude signal analysis technique, as illustrated in Figs 8 and 9. The regions of concentrated energy (amplitude) correspond temporally to the moments the roller passes the sensor and spectrally to the 20–40 Hz frequency range. In the X-direction, the energy concentration exhibits a wider temporal bandwidth. As the number of rolling passes increases, the dominant frequency initially rises before stabilizing; meanwhile, the peak energy increases over the first three passes but declines at the 4th pass. Notably, high-frequency energy signals (>40 Hz) are predominantly present during the 2nd and 3rd passes. In contrast, the Z-direction displays a narrower temporal energy band, which gradually widens with successive passes, while the prevalence of high-frequency energy signals progressively diminishes.

**Fig 8:**
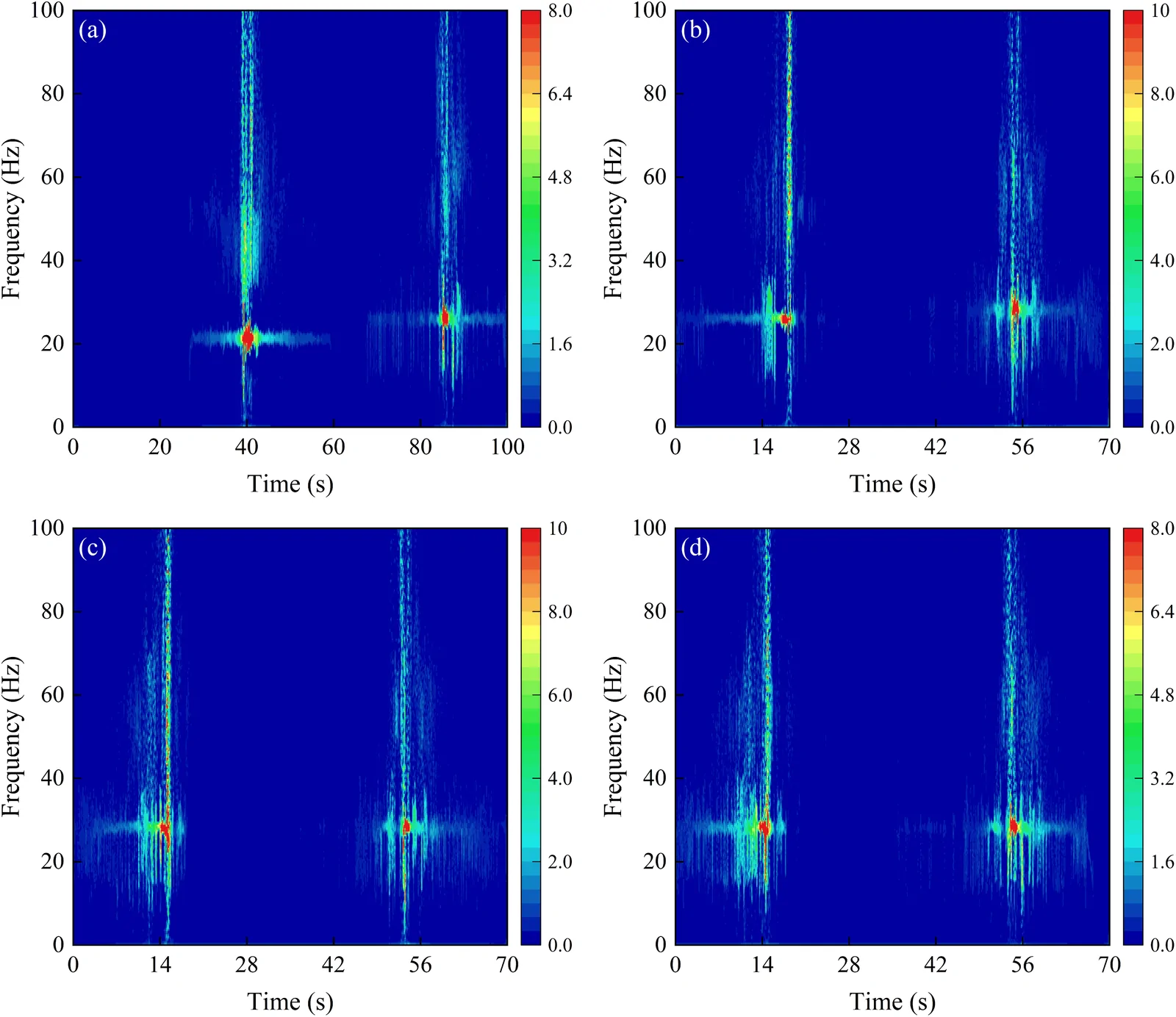
Hilbert spectrum in the X-direction of the subgrade under (a) 1 pass, (b) 2 passes, (c) 3 passes, and (d) 4 passes of roller compaction.

**Fig 9:**
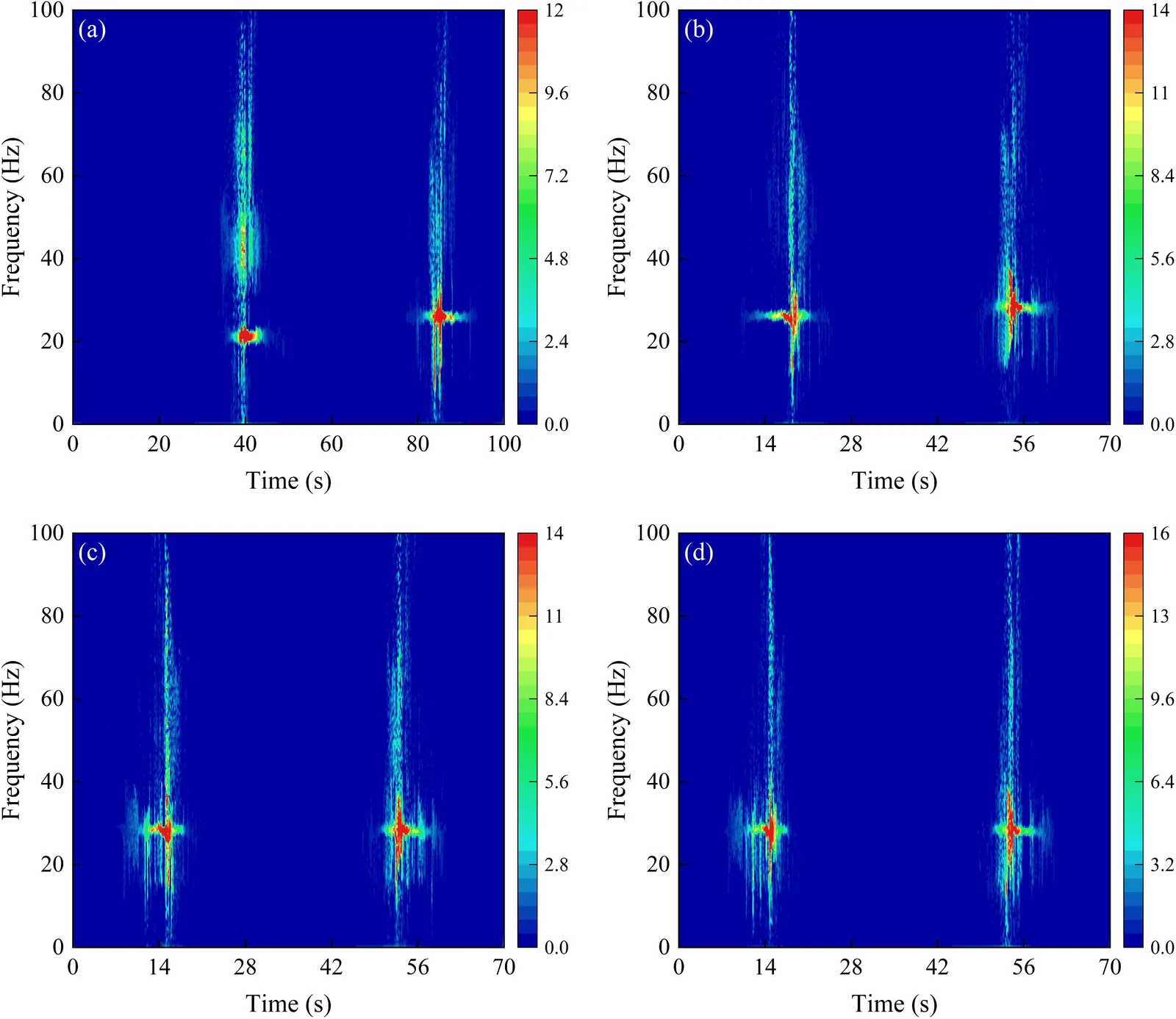
Hilbert spectrum in the Z-direction of the subgrade under (a) 1 pass, (b) 2 passes, (c) 3 passes, and (d) 4 passes of roller compaction.

During the early stages of compaction, complex shear and surface interactions resulting from material inhomogeneity lead to relatively dispersed energy transmission. The abundance of high-frequency components during the 3rd pass likely indicates that the material’s internal structural adjustment has reached an active peak. Conversely, the subsequent energy decrease during the 4th pass suggests that shear characteristics may be degrading due to over-compaction. Overall, these energy evolution characteristics collectively corroborate the existence of an optimal state in the compaction process (approximately at the 3rd pass), where energy transmission is most efficient and concentrated. The attenuation of energy in the 4th pass implies that continued rolling has entered a stage of diminishing returns, potentially triggering subtle degradation in material performance.

#### 3.2.2 Energy attenuation mechanism.

To elucidate the energy attenuation mechanism of roller-induced vibration waves along the horizontal path, normalized instantaneous peak acceleration values were extracted from five measurement points located at the same depth as the vibratory roller reached point A18 (Fig 10). The vibration waves propagate outward from point A18, exhibiting rapid attenuation with increasing distance. Regarding particle motion, in the X-direction, normalized amplitudes range from 0.11 to 0.19 at a distance of 2 m, and from 0.02 to 0.14 at 4 m. Comparatively, amplitudes of the Z-direction particle motion are lower, ranging from 0.05 to 0.14 at 2 m and 0.01 to 0.07 at 4 m. Furthermore, as the number of rolling passes increases, the normalized amplitude in the X-direction rises significantly, whereas the Z-direction exhibits an inverse trend.

**Fig 10:**
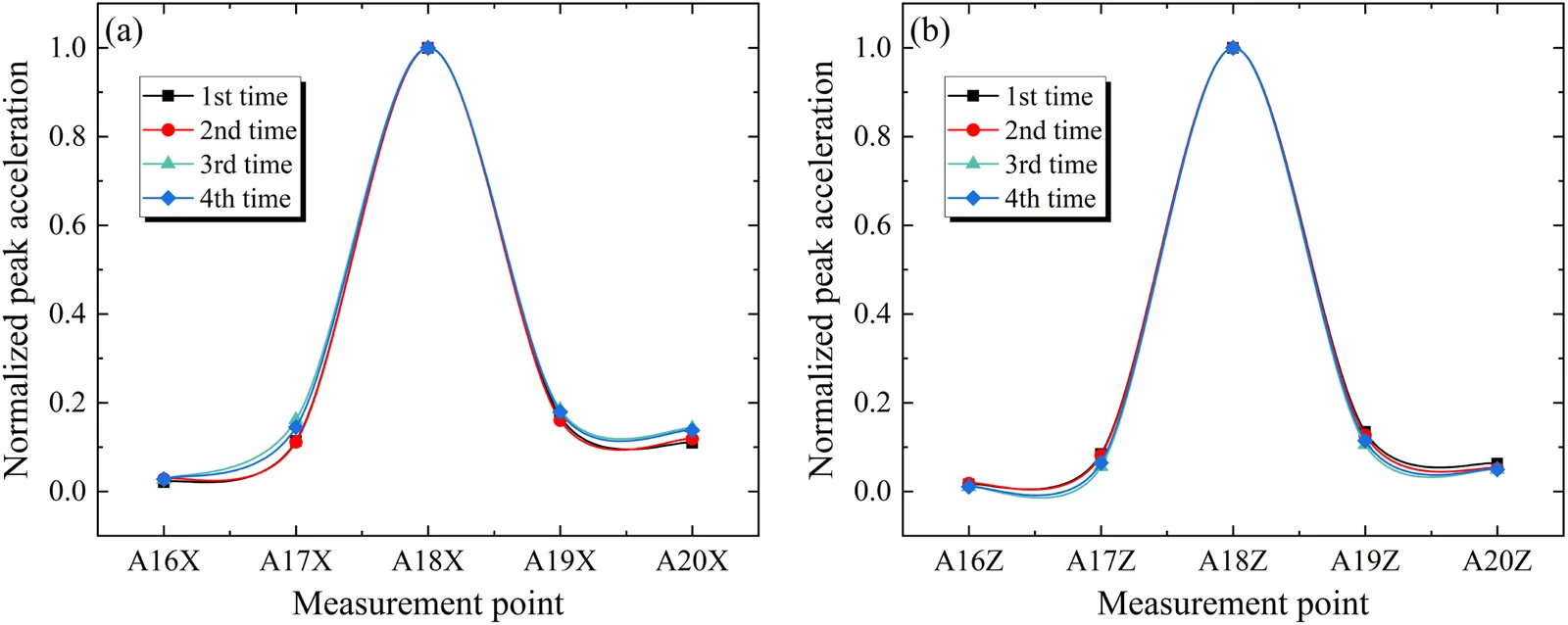
Normalized peak accelerations of the subgrade during roller compaction in the (a) X direction and (b) Z direction.

Significant energy dissipation occurs along the horizontal propagation path. The rapid spatial decay of the Z-direction motion suggests that the vertical excitation energy is primarily consumed as compaction work in the immediate near-field of the roller. In contrast, the X-direction motion likely comprises surface wave components; their attenuation is relatively slower, facilitating detection at greater distances. As the number of rolling passes increases, material density and stiffness improve, creating a more uniform and consolidated medium. This evolution optimizes the horizontal wave transmission path, reducing energy loss from scattering caused by inhomogeneity. Consequently, the high Z-axis response remains strongly confined to the localized area near the vibration source, rather than radiating outward horizontally. This enhanced near-field localization of energy transfer indicates improved compaction efficiency and the material’s transition toward uniformity and elasticity.

### 3.3 Compactness of the subgrade

Upon completion of the designated rolling scenario for each test lane, the sand cone method was employed to measure the actual compactness near the acceleration measurement points. Using the central measurement point as a representative example, the relationship between the compactness and the number of rolling passes is illustrated in Fig 11. The data indicate degrees of compaction of 88.78%, 92.37%, 96.72%, and 95.77% after the first, second, third, and fourth passes, respectively. When synthesized with the preceding acceleration analysis, these results confirm that three passes constitute the optimal compaction effort for this fill material. Beyond this point, additional input energy is no longer utilized for effective densification; instead, it induces micro-damage within the established structural skeleton, resulting in a slight reduction in compaction degree and an inflection point in the dynamic properties. By correlating real-time dynamic monitoring with traditional quality inspection, this study demonstrates that dynamic indices can serve as real-time, non-destructive “precursor signals,” effectively providing early warning of over-compaction risks before traditional methods detect a decline in quality.

**Fig 11:**
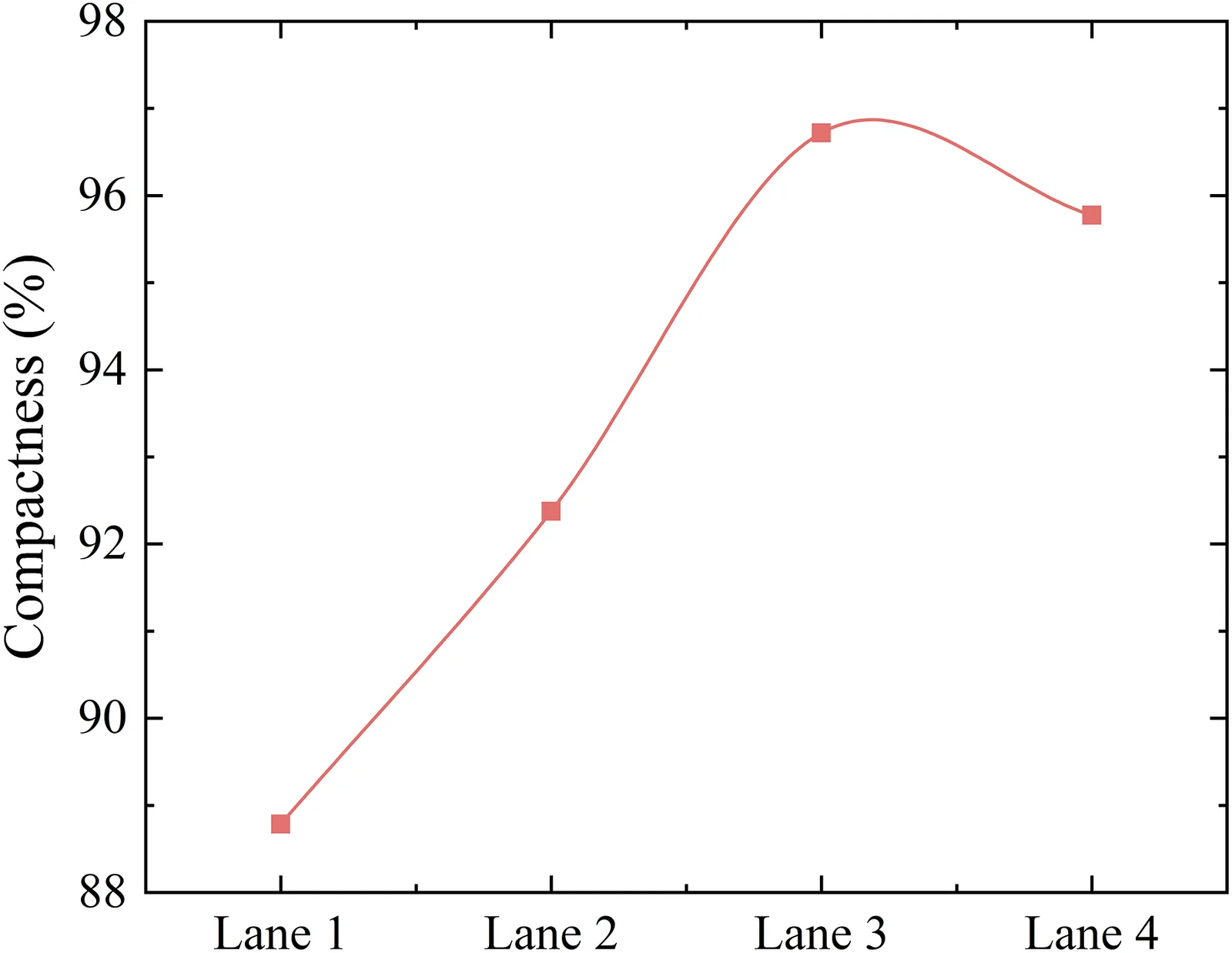
Compactness of subgrade under different numbers of rolling passes.

### 3.4 Compaction efficiency and energy transfer

During vibratory rolling, the energy transfer between the vibratory drum and the subgrade is critical. To address this, this study introduces a novel compaction efficiency evaluation method based on an energy transfer coefficient, established through synchronous monitoring of the acceleration responses of both the vibratory drum and the subgrade. Time windows corresponding to the roller’s passage directly over each ground sensor were identified, allowing for the extraction of the respective drum and ground acceleration signals for analysis (Fig 12). Root Mean Square (RMS) values for both accelerations were calculated within these windows to quantify vibration intensity. The energy transfer coefficient, denoted as *η*, is defined as the ratio of the ground acceleration RMS value to the drum acceleration RMS value. This dimensionless parameter quantifies the proportion of input energy converted into a vibration response within the soil at a specific measurement point.

**Fig 12:**
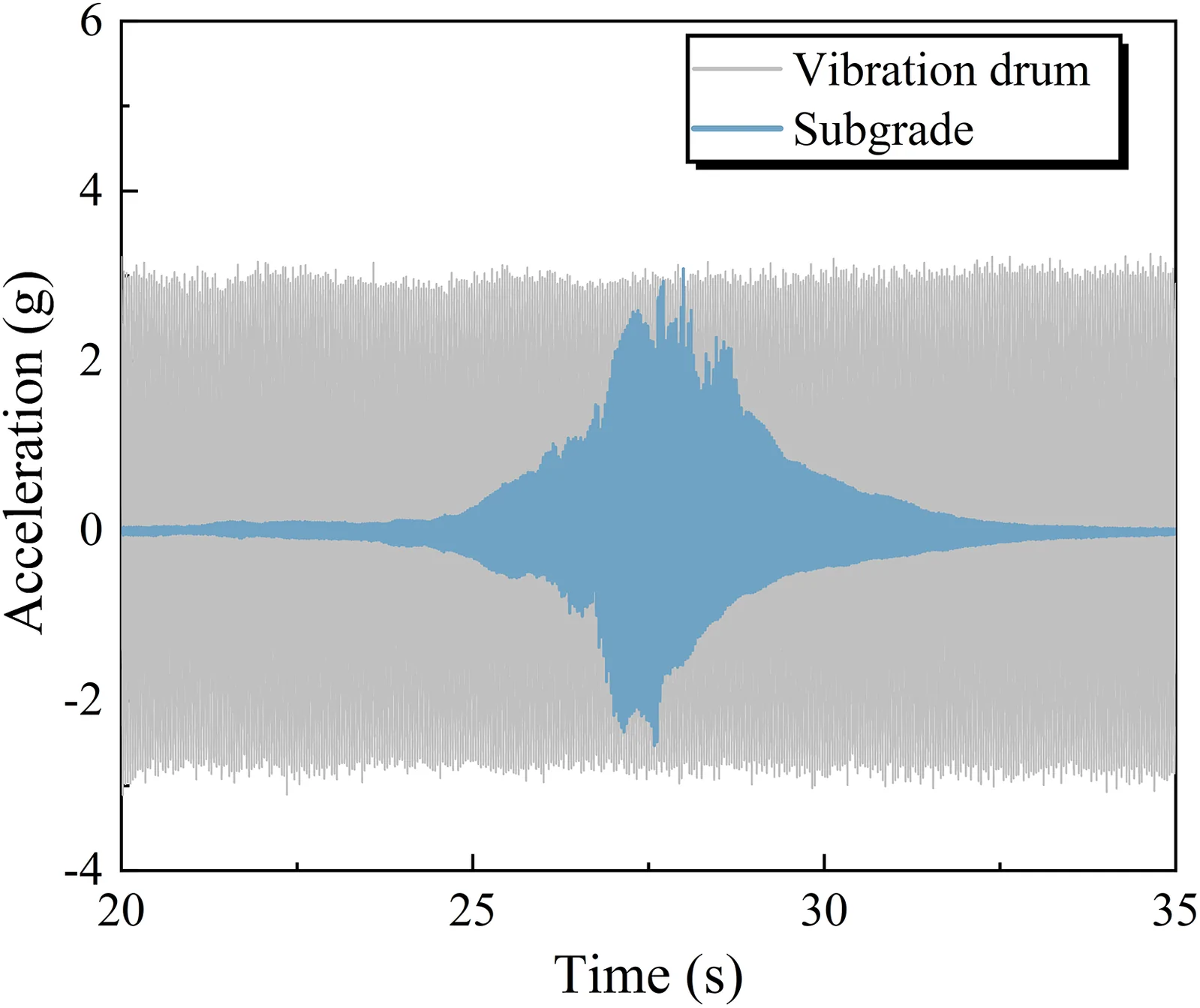
The time window during which the roller passes over the ground sensors.

The energy transfer coefficients *η* corresponding to different rolling passes are presented in Table 3. During the first pass, the soil remains in a loose state; consequently, significant energy is consumed to overcome inter-particle friction and fill voids, resulting in permanent deformation. This leads to a weak ground response and a relatively low *η* value. During the second and third passes, the soil densifies and its elasticity increases. Input energy propagates more effectively through the medium as elastic waves, inducing strong vibrations that facilitate further densification. During this phase, *η* rises sharply. However, after the fourth pass, as the soil approaches its optimal compactness and stiffness increases, the energy transfer coefficient exhibits a slight decline. This reduction is attributed to the reflection of energy by the stiffened soil and dissipation through ineffective impacts.

**Table 3:** Energy transfer coefficient *η.*

	1st rolling pass	2nd rolling pass	3rd rolling pass	4th rolling pass
Condition 1	0.240	/	/	/
Condition 2	0.253	0.260	/	/
Condition 3	0.233	0.250	0.252	/
Condition 4	0.249	0.255	0.252	0.243

## 4 Discussion

Vibratory compaction of highway subgrades is not a simple linear densification process. Instead, it is a dynamic process characterized by evolving internal structure and systematic changes in wave propagation characteristics [18,40]. Existing studies on vibratory compaction monitoring have predominantly focused on isolated, retrospective comparisons between dynamic indicators and final compaction quality [27,28]. This approach, however, fails to reveal the intrinsic, coupled evolution of multi-dimensional dynamic characteristics (e.g., time-domain responses, frequency spectra, energy dissipation, and spatial attenuation) during compaction. Consequently, a comprehensive framework linking vibration signals to evolving material structural states cannot be constructed. Furthermore, real-time, non-destructive precursors or criteria for identifying the critical state of over-compaction are lacking. As a result, construction control continues to rely on post-construction inspection. This prevents proactive termination of operations at optimal compaction quality, leading to risks of performance degradation and resource waste.

Building on this premise, this study utilizes multi-dimensional signals from field subgrade compaction tests to characterize time-domain acceleration peaks, spectral structures, time-frequency energy distributions, and spatial wave attenuation patterns. These dynamic characteristics are then correlated with final compactness measurements through coupled analysis. The effectiveness of the compaction process manifests through distinct dynamic signatures. Specifically, from the first to the third pass, compactness increased steadily from 88.78% to 96.72%. Concurrently, the dynamic system transitioned from a high-dissipation, non-linear state to a low-damping, linear state. Regarding energy propagation, vibration waves in the X-direction propagated further as the number of rolling passes increased, indicating an increase in the material’s shear modulus and enhanced wave-guiding capabilities. These observations confirm that effective compaction not only improves static compactness but also fundamentally reconstructs the material’s dynamic constitutive relationship, transforming it into a more efficient medium for vibration energy transmission.

Furthermore, dynamic responses sensitively capture the accumulation of internal material damage before traditional compactness detection methods can confirm performance degradation. Such damage, potentially stemming from aggregate crushing or the formation of microscopic shear bands, leads to macroscopic stiffness degradation. This degradation causes the system to deviate from its resonance state and alters energy dissipation mechanisms. Consequently, the inflection point of the Z-direction acceleration peak, the reduction in dominant frequency amplitude, and the rapid attenuation of the X-direction response serve as real-time criteria for identifying the onset of over-compaction and determining the “optimal number of rolling passes”.

Finally, the proposed compaction efficiency evaluation method based on the energy transfer coefficient not only reveals, from a fundamental physical perspective, the dynamic mechanism of the transition from plastic deformation to elastic response during the compaction process. Crucially, it provides a quantitative criterion based on energy efficiency to resolve the engineering challenge of “when to stop rolling” inherent in traditional compaction quality control. Analysis indicates that compaction effectiveness stabilizes when the energy transfer coefficient reaches a plateau; this point corresponds to the economically optimal number of rolling passes.

Despite the rigorous experimental design, this study is subject to certain limitations. As the conclusions are predicated on specific backfill materials and operational conditions, the derived optimal pass counts, dominant frequency characteristics, and attenuation patterns may lack generalizability to other geomaterials or excitation modes. Additionally, the magnitude of the energy transfer coefficient within the proposed evaluation method is sensitive to the selection of the data time window. Specifically, a narrower window length exacerbates the numerical variance in the energy transfer coefficient, whereas an excessively short window risks the loss of critical data. Consequently, additional field testing and data fitting are necessary to strike an optimal balance, thereby enhancing the broader applicability of this evaluation metric. Future research should focus on developing generalized dynamic models that account for varying material types, moisture contents, and excitation parameters. Furthermore, establishing digital correlations between these dynamic criteria and traditional indices, such as compactness and stiffness modulus, will be essential for advancing autonomous compaction decision-making systems.

## 5 Conclusions

Field tests were conducted to investigate how the number of rolling passes influences the compaction quality of a highway soil-rock mixture subgrade. The study analyzed the intrinsic coupled evolution of the subgrade’s multi-dimensional dynamic characteristics under low-amplitude excitation. The primary conclusions are as follows:

(1) The subgrade material undergoes a systematic dynamic transition, categorized into three stages: loose, optimal compaction, and over-compaction. The effective compaction stage is characterized by a significant increase in the Z-direction acceleration peak, attributed to increased system stiffness and the approach to resonance; a spectral evolution from disordered broadband noise to a regular harmonic structure centered on the fundamental excitation frequency; and enhanced vibration wave propagation in the X-direction.
(2) A robust correlation was established between the dynamic response and final compaction quality, allowing for the determination of the optimal compaction state. Under the conditions of this test, the third rolling pass corresponded to the comprehensive optimal dynamic state (characterized by resonance peaks, a regular spectrum, and efficient energy transfer) and the highest compactness; this was defined as the optimal number of rolling passes.
(3) Effective compaction optimizes the subgrade’s wave-guiding properties. As compactness increases, the horizontal propagation of X-direction particle motion improves, while Z-direction motion attenuates rapidly along the horizontal path. This highly localized Z-direction response indicates that vertical excitation energy is effectively confined to the near-field for compaction, rather than dissipating laterally.
(4) A compaction efficiency evaluation method based on the energy transfer coefficient was developed to elucidate energy utilization dynamics. This method validates the three-stage compaction process from an energetic perspective and provides a quantitative indicator for real-time quality assessment and the determination of the compaction endpoint.

## Supporting information

S1 Dataset Source Data for Figures 3–12. (XLSX)
